# Estrogen Modulates the Sensitivity of Lung Vagal C Fibers in Female Rats Exposed to Intermittent Hypoxia

**DOI:** 10.3389/fphys.2018.00847

**Published:** 2018-07-05

**Authors:** Ya-Chen Huang, Zung Fan Yuan, Chang-Huan Yang, Yan-Jhih Shen, Jyun-Yi Lin, Ching Jung Lai

**Affiliations:** ^1^Department of Chest Section, Buddhist Tzu Chi General Hospital, Hualien City, Taiwan; ^2^Master Program in Physiological and Anatomical Medicine, School of Medicine, Tzu Chi University, Hualien City, Taiwan; ^3^Department of Physiology, Tzu Chi University, Hualien City, Taiwan; ^4^Institute of Physiology, National Yang-Ming University, Taipei, Taiwan; ^5^Ph.D. Program in Pharmacology and Toxicology, School of Medicine, Tzu Chi University, Hualien, Taiwan

**Keywords:** intermittent hypoxia, airway hypersensitivity, lung vagal C fibers, estrogen, lung inflammation

## Abstract

Obstructive sleep apnea is mainly characterized by intermittent hypoxia (IH), which is associated with hyperreactive airway diseases and lung inflammation. Sensitization of lung vagal C fibers (LVCFs) induced by inflammatory mediators may play a central role in the pathogenesis of airway hypersensitivity. In females, estrogen interferes with inflammatory signaling pathways that may modulate airway hyperreactivity. In this study, we investigated the effects of IH on the reflex and afferent responses of LVCFs to chemical stimulants and lung inflammation in adult female rats, as well as the role of estrogen in these responses. Intact and ovariectomized (OVX) female rats were exposed to room air (RA) or IH for 14 consecutive days. On day 15, IH enhanced apneic responses to right atrial injection of chemical stimulants of LVCFs (e.g., capsaicin, phenylbiguanide, and α,β-methylene-ATP) in intact anesthetized females. Rats subjected to OVX prior to IH exposure exhibited an augmented apneic response to the same dose of stimulants compared with rats subjected to other treatments. Apneic responses to the stimulants were completely abrogated by bilateral vagotomy or perivagal capsaicin treatment, which blocked the neural conduction of LVCFs. Electrophysiological experiments revealed that in IH-exposed rats, OVX potentiated the excitability of LVCFs to stimulants. Moreover, LVCF hypersensitivity in rats subjected to OVX prior to IH exposure was accompanied by enhanced lung inflammation, which was reflected by elevated inflammatory cell infiltration in bronchoalveolar lavage fluid, lung lipid peroxidation, and protein expression of inflammatory cytokines. Supplementation with 17β-estradiol (E2) at a low concentration (30 μg/ml) but not at high concentrations (50 and 150 μg/ml) prevented the augmenting effects of OVX on LVCF sensitivity and lung inflammation caused by IH. These results suggest that ovarian hormones prevent the enhancement of LVCF sensitivity and lung inflammation by IH in female rats, which are related to the effect of low-dose estrogen.

## Introduction

Airway exposure to long-term intermittent hypoxia (IH) may contribute to several obstructive sleep apnea (OSA)-related hyperreactive airway diseases such as asthma (Teodorescu et al., 2012), chronic cough (Chan et al., 2015), and bronchial hyperreactivity (Sariman et al., 2011). Previous studies have reported that increased airway levels of inflammation and oxidative stress were found in patients with OSA (Aihara et al., 2013; Tichanon et al., 2016) and IH-exposed animals (Yang et al., 2016a,b). Premenopausal women have a lower prevalence and milder severity of OSA than men; however, post-menopausal women have the same incidence rate as men (Nieto et al., 2000; Jordan and McEvoy, 2003; Resta et al., 2003). Sex hormones, especially estrogen, play an important role in responses and adaptations to hypoxic exposure (McMurtry et al., 1973; Stupfel et al., 1984; Ou et al., 1994). In addition, estrogen can modulate the immune function of various cell types (Straub, 2007) and possibly interferes with the production of various cytokines, such as interleukin-1β (IL-1β), NF-κB, and tumor necrosis factor-α (TNF-α) in several systems (Stein and Yang, 1995; Cerillo et al., 1998; Evans et al., 2002; Sarvari et al., 2011). Although estrogen is a critical contributor to gender effects on OSA-associated cardiovascular consequences (Kapsimalis and Kryger, 2002; Lan et al., 2017), its role in the development of OSA-related hyperreactive airway diseases in females remains unclear.

Airway hypersensitivity, manifested by exaggerated airway reflex responses to stimuli, is due to sensitization of lung afferents under inflammatory conditions (Lee, 2009; Kou et al., 2011). Activation of lung vagal C fibers (LVCFs), the dominant type of lung afferent fibers, elicits a number of airway reflexes, including apnea, cough, mucus secretion, and airway constriction (Lee, 2009). A growing body of evidence indicates that the sensitivity of LVCFs can be enhanced by lung inflammation and has a major pathophysiological role in hyperreactive airway diseases (Lee, 2009). IH exposure triggers inflammatory responses by promoting the release of various inflammatory mediators in the lungs (Yang et al., 2016a; Lee et al., 2017). Many of these mediators can sensitize LVCFs and consequently lead to the development of airway hypersensitivity (Ho et al., 2000; Ruan et al., 2014). We recently reported that 14 days of IH exposure can evoke LVCF-mediated airway hypersensitivity in male rats and that the production of reactive oxygen species (ROS) is responsible for the sensitizing effect of IH (Yang et al., 2016b). However, whether LVCF-mediated airway hypersensitivity is induced in female rats after 14 days of IH exposure remains unknown.

Exacerbated airway inflammation under IH exposure is a major contributor to the development of airway hypersensitivity (Yang et al., 2016a). Estrogen has diverse modulatory effects on the inflammatory cascade. These effects are dependent on the stimuli and target organ, as well as on estrogen receptor expression, estrogen level fluctuations, and estrogen concentration (Straub, 2007). For example, ovariectomy (OVX) increases the infiltration of inflammatory cells into the lungs after ovalbumin challenge; the subcutaneous injection of 17β-estradiol (E2), the most potent and predominant estrogen, can prevent excessive cell infiltration (de Oliveira et al., 2010). Conversely, E2 can also promote the expression and production of numerous inflammatory cytokines in lipopolysaccharide-activated macrophages (Calippe et al., 2008) and in thioglycolate-elicited macrophages (Calippe et al., 2010). Different doses of E2 may exert diverse effects on inflammatory reactions (Straub, 2007; Sarkaki et al., 2013). Inflammatory and immune reactions may be exacerbated by a physiologically high concentration of E2 (Hirano et al., 2007) but may be attenuated by low doses of E2 (Straub, 2007; Samantaray et al., 2016). However, whether E2 modulates IH-induced airway hypersensitivity in female rats remains unknown.

This study aimed to investigate whether 14 days of exposure to IH enhances the reflex and afferent responses of LVCFs to chemical stimulants in female rats; whether OVX-augmented LVCF sensitivity induced by IH is associated with lung inflammation; and whether the supplementation of physiological E2 concentrations influences LVCF hypersensitivity and lung inflammation in OVX rats exposed to IH. To accomplish these objectives, the reflex and afferent responses to LVCF stimulants in intact and OVX female rats treated with different doses of E2 were studied. These stimulants included capsaicin (Yang et al., 2016b), phenylbiguanide (Yang et al., 2016a), and α,β-methylene-ATP (Yang et al., 2016a).

## Materials and Methods

### Animals and Pretreatment

Experiments were performed on female adult Sprague-Dawley rats (10–12 weeks of age). Experimental procedures were approved by the Institutional Animal Care and Use Committee of Tzu Chi University. Some female rats were anesthetized with Zoletil 50 (40 mg/kg, i.p.; Virbac, Carros, France) for OVX and allowed to recover for 7 days prior to RA or IH exposure. Silicone capsules (3.18 mm o.d. × 1.57 mm i.d., 20 mm active length, s.c.; A-M System, Carlsborg, WA, United States) containing E2 (0, 30, 50, and 150 μg/ml corn oil; Sigma-Aldrich, St. Louis, MO, United States) were subcutaneously implanted in separate groups of OVX rats for five additional days prior to IH exposure. These capsules can provide a sustained level of plasma E2 in a reliable range depending on concentration gradient (Goodman, 1978).

### Exposure to IH

A rat model of IH was established in accordance with a previous method (Lai et al., 2006). Pure nitrogen was allowed to enter the Plexiglas cylindrical chamber for 30 s through a timed solenoid valve with flow regulators. The inspired O_2_ fraction (FIO_2_) nadir in the chamber was adjusted to 5% for 2–5 s. Compressed air was then infused for 45 s to allow the FIO_2_ to return gradually to 20.9%. Rats were exposed to IH for 6 h (10:00–16:00) per day for 14 consecutive days. Rats in the RA control group were exposed to alternating cycles of compressed air for 14 days. After the daily exposure procedure, all rats were placed individually in clear acrylic chambers.

### Animal Preparation

Sixteen hours after the last exposure to RA or IH, rats were anesthetized through the intraperitoneal injection of α-chloralose (100 mg/kg; Sigma-Aldrich) and urethane (500 mg/kg; Sigma-Aldrich) dissolved in a borax solution (2%; Sigma-Aldrich). Supplemental doses were administered intravenously to abolish pain reflexes. The trachea was then cannulated below the larynx using a short tracheal tube *via* a tracheotomy. The right jugular vein and right femoral artery were cannulated for the administration of chemicals and the measurement of arterial blood pressure (ABP), respectively. Body temperature was maintained at approximately 36°C by a servo-controlled heating blanket. The animals were sacrificed at the end of experiments through an overdose of intravenously administered anesthetic.

### Determination of Estrus Stages

Estrus stages were determined on the basis of vaginal smear prepared with saline. Cell types of specimens were examined under the microscope prior to the measurements of ventilatory responses or afferent activities of LVCF. Estrus stages were determined using a method described in detail previously (Ji et al., 2008).

### Measurement of Ventilatory Responses

In reflex studies, the rats were allowed to breath spontaneously through the tracheal cannula. Respiratory flow (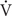
R) was measured with a pneumotachograph (Fleisch 4/0; Richmond, VA, United States) coupled to a differential pressure transducer (Validyne MP45-12), and the results were integrated to obtain tidal volume (VT). Tracheal pressure (Ptr) was recorded by using a pressure transducer (Validyne MP45-28) through a side tap to the tracheal cannula.

### Perivagal Capsaicin Treatment

To selectively block the neural conduction of LVCFs, bilateral cervical vagus nerves were subjected to perivagal capsaicin treatment in accordance with a previously described method (Kou et al., 1995). Cotton strips were soaked in capsaicin solution (250 μg/ml; Sigma) and wrapped around a 2- to 3-mm segment of isolated cervical vagus nerves. After 20 min, when the apneic reflex response to the intravenous injection of capsaicin (1 μg/kg) was abolished, the cotton strips were removed.

### Recording of Afferent Activity

In electrophysiological studies, afferent activities arising from LVCFs were recorded in open-chest artificially ventilated rats by using techniques described elsewhere (Lai and Kou, 1998). Lungs were ventilated by a respirator (Harvard 683; South Natick, MA, United States), and VT and respiratory rate were set at 7–8 ml/kg and 60 breaths/min, respectively. A midline thoracotomy was performed and the expiratory outlet of the respirator was placed under 3 cmH_2_O pressure to maintain a near-normal functional residual capacity. During the recording of LVCF activities, rats were paralyzed with pancuronium bromide (0.05 mg/kg, i.v.; Organon Teknika, Boxtel, Holland). Briefly, the afferent activities of LVCFs were searched for initially by their mild response to lung hyperinflation (three to four times VT) and by an immediate (within 2 s) response to a bolus injection of capsaicin (1 μg/kg), a potent chemical stimulant of LVCFs, into the right atrium. The conduction velocity of the afferent fibers of the receptors was measured through a previously described method (Ho et al., 2001). At the end of each experiment, the general locations of LVCFs were identified on the basis of their responses to the gentle pressing of the lungs with a saline-moistened cotton Q-tip.

### Preparation of Bronchoalveolar Lavage Fluid

At the end of each experiment, the lungs of rats were intratracheally lavaged thrice with 3 ml of saline containing protease inhibitor cocktail set III (Calbiochem, San Diego, CA, United States). Bronchoalveolar lavage fluid (BALF) samples were then centrifuged at 1200 × *g* for 10 min at 4°C. Supernatant samples were maintained at -80°C for subsequent analysis. Cell pellets from BALF samples were resuspended in PBS for cell counting. Differential leukocyte counts were performed on cytospin preparations in accordance with standard morphologic criteria.

### Enzyme-Linked Immunosorbent Assay

At the end of measurements of ventilatory responses, arterial blood samples were collected through the cannulation of the right femoral artery. Serum was isolated and frozen at -80°C. Serum estradiol levels were analyzed by an enzyme-linked immunosorbent assay (ELISA) kit (Calbiotech, Spring Valley, CA, United States). TNF-α levels in BALF supernatant were measured using an ELISA kit (R&D systems, Minneapolis, MN, United States).

### Western Blot Analysis

Lung tissues were collected from sacrificed animals and stored at -80°C. To quantify the expression levels of nuclear NF-κB-p65, IL-1β, and COX-2 in lung tissues, frozen lung tissues were homogenized by using a tissue grinder in permeabilization buffer containing protease inhibitors (iNtRON Biotechnology, Korea). Nuclear proteins were extracted in accordance with a previously reported method (Xu and Stern, 2003) with slight modifications. Samples were separated in 10% SDS–PAGE and then transferred to polyvinylidene fluoride membranes (Merck Millipore Corporation, United States). The membranes were blocked with 5% skim milk and incubated with different primary antibodies and their corresponding secondary antibodies. The following primary antibodies were used: mouse anti-NF-κB-p65 (1:2000; Cat. # 6956S, Cell Signaling), rabbit anti-IL-1β (1:1000; Cat. # ab9787, Abcam), and rabbit anti-COX-2 (1:1000; Cat. # 4842S, Cell Signaling). Specific protein bands were detected by using an enhanced chemiluminescence kit (GE Healthcare, United States). Blots were visualized by exposing the membranes to X-ray film (Kodak, United States) and quantified using ImageJ processing software.

### Measurement of Lung Oxidative Stress

The concentrations of malondialdehyde (MDA), a product of lipid peroxidation and a biomarker of oxidative stress, were measured by thiobarbituric acid assay kit (Cayman, Ann Arbor, MI, United States) according to the manufacturer’s instructions.

### Pharmacological Agents

To stimulate LVCFs, capsaicin (1 μg/kg), a transient receptor potential vanilloid 1 receptor agonist (Shen et al., 2012), phenylbiguanide (8 μg/kg), a 5-HT3 receptor agonist) (Yang et al., 2016a), and α,β-methylene-ATP (15 μg/kg in the reflex studies and 100 μg/kg in the electrophysiological studies), a P2X receptor agonist (Yang et al., 2016a), were injected as a bolus into the right atrium (volume 0.1 ml) and flushed by injection with 0.3 ml of saline. Saline was used as the vehicle for phenylbiguanide and α,β-methylene-ATP. A solution containing 10% Tween 80, 10% ethanol, and 80% saline was used as the vehicle for capsaicin.

### Experimental Design and Protocols

Four series of experiments were conducted in this study and involved 178 female rats that were divided into 21 study groups. All groups consisted of 10 rats each, except in each group that corresponded to each of the four stages of the estrus cycle comprised six rats. The apneic reflex responses and afferent responses of LVCFs to injections of the three chemical stimulants (capsaicin, phenylbiguanide, and α,β-methylene-ATP) were measured. An interval of ∼15 min was allowed between any two injections of stimulants to allow the baseline respiratory pattern or fiber activity (FA) to return to its control level. Only one LVCF was recorded from each animal. In study series 1, the apneic reflex responses and afferent responses of LVCFs to three stimulants were studied in intact female rats exposed to RA for 14 days over the four stages of the estrus cycle: diestrus (*Groups 1 and 2*), proestrus (*Groups 3 and 4*), estrus (*Groups 5 and 6*), and metestrus (*Groups 7 and 8*). This experiment was performed to study the influence of estrus cycle. In study series 2, the apneic reflex responses and afferent responses of LVCFs to the stimulants were assessed in intact female rats exposed to RA (RA; *Groups 1–8*) or IH (IH; *Groups 9 and 10*) and OVX rats exposed to IH (OVX + IH; *Groups 11 and 12*). This experiment was performed to assess the potentiating effect of IH and the role of ovarian hormones. To determine the role of the LVCFs in the apneic responses to the stimulants, C-fiber function was subsequently blocked through perivagal capsaicin treatment and bilateral cervical vagotomy. In addition, the apneic reflex responses to the stimulants in OVX rats exposed to RA (OVX + RA; *Group 13*) were also evaluated to identify the role of endogenous ovarian hormones in the development of airway sensitivity under normal conditions. In study series 3, the apneic reflex responses and afferent responses of LVCFs to the stimulants were investigated in OVX rats exposed to IH for 14 days and supplemented with the vehicle (Veh + OVX + IH; *Groups 14 and 15*) or three different concentrations of E2: 30 μg/ml (30E2 + OVX + IH; *Groups 16 and 17*), 50 μg/ml (50E2 + OVX + IH; *Groups 18 and 19*), and 150 μg/ml corn oil (150E2 + OVX + IH; *Groups 20 and 21*). This experiment aimed to assess the role of estrogen in the responses to IH. In study series 4, arterial blood samples were obtained from *Groups 1, 3, 5, 7, 9, 11, 14, 16, 18, and 20* (RA or IH in reflex studies) for the measurement of estradiol concentration. BALF samples were also obtained for the measurement of inflammatory cells and TNF-α levels in these groups. In study series 5, lung tissues were obtained for the measurement of the protein levels of NF-κB, IL-1β, and COX-2, as well as MDA concentration from *Groups 2, 4, 6, 8, 10, 12, 17, 19*, and *21* (RA or IH in afferent studies).

### Data Analysis and Statistics

Respiratory frequency (*f*), expiratory duration (TE), and VT were analyzed on a breath-by-breath basis as the average value over the 10-breath period prior to chemical stimulation. The longest TE that occurred during the first 20 s after stimulant injection was divided by the baseline TE to give a percentage apneic ratio for the comparison of apneic responses induced by various experimental conditions. Baseline FA was calculated as the average value over a 10-s interval immediately before chemical stimulation. Peak responses were defined as the maximum average over a 2-s interval during the 20 s following the injection of the stimulant. Mean ABP and heart rate (HR) were continuously analyzed at 1-s intervals. All physiological parameters were analyzed using a computer equipped with an analog-to-digital converter (Gould DASA 4600) and software (BioCybernatics 1.0, Taipei, Taiwan). Data were compared using either one-way ANOVA or two-way mixed factorial ANOVA, followed by Neuman–Keuls tests when appropriate. Differences were considered statistically significant at *p* < 0.05. All data are reported as means ± SE.

## Results

### Baseline Physiological Parameters

The results of reflex studies revealed no significant differences among the baseline respiratory variables, including *f*, VT, and TE of RA, IH, OVX + RA, and OVX + IH rats. In contrast to vehicle supplementation, E2 supplementation at three different concentrations (30, 50, and 150 μg/ml) did not affect the averages of the baseline respiratory parameters of OVX + IH rats. In the electrophysiological studies, 100 LVCF fibers were used to responses to the chemical stimulants. The baseline LVCF activities of OVX + IH rats (0.08 ± 0.03 impulses/s; *n* = 10) and IH rats (0.05 ± 0.02 impulses/s; *n* = 10) were not significantly different from those of RA rats (0.04 ± 0.01 impulses/s; *n* = 24). In contrast to those in Veh + OVX + IH rats, the mean baseline respiratory variables and the baseline LVCF activity were also not significantly altered by E2 replacement at three different concentrations in OVX + IH rats. The mean conduction velocities of 80 LVCFs studied were 1.10 ± 0.05 m/s (range 0.73–1.91 m/s). These LVCFs were all localized within lung structures. The mean ABP (128.9 ± 4.0 mmHg) but not the HR (374.9 ± 13.2 beats/min) of OVX + IH rats was significantly greater than that of RA rats (mean ABP = 106.8 ± 2.5 mmHg; HR = 323.4 ± 8.4 beats/min), IH rats (mean ABP = 115.6 ± 2.5 mmHg; HR = 326.7 ± 15.0 beats/min), and RA + OVX rats (mean ABP = 108.8 ± 3.0 mmHg; HR = 342.4 ± 11.9 beats/min). In addition, supplementation with E2 at a low concentration (30 μg/ml; mean ABP = 114.1 ± 3.4 mmHg) but not at high concentrations (50 μg/ml; mean ABP = 121.9 ± 3.9 mmHg and 150 μg/ml; mean ABP = 121.5 ± 4.8 mmHg) prevented the potentiating effect of OVX on the mean ABP in IH-exposed rats. By contrast, supplementation with the vehicle (mean ABP = 126.5 ± 5.3 mmHg) failed to produce this effect.

### Influence of Estrus Stages on the Apneic Reflex Responses and Afferent Responses of LVCFs to Chemical Stimulants in Intact Female Rats

**FIGURE 1:**
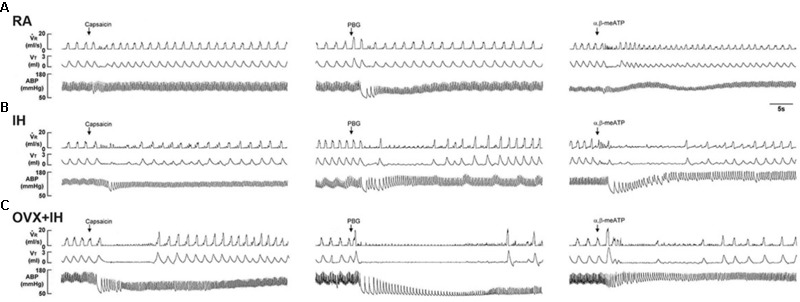
Ventilatory responses to the intravenous injection of three types of stimulants in three female rats after 14 days of exposure to room air (RA) or intermittent hypoxia (IH). **(A)** Responses of an intact rat exposed to RA; **(B)** responses of an intact rat exposed to IH; **(C)** responses of an ovariectomized (OVX) rat exposed to IH (OVX + IH). Sixteen hours after the last exposure, the animals’ responses to capsaicin (1.0 μg/kg), phenylbiguanide (PBG; 8.0 μg/kg), and α,β-methylene-ATP (α,β-meATP; 10 μg/kg) were measured in each rat. These chemicals are stimulants of lung vagal C fibers and were injected into the jugular vein as a bolus (0.1 ml volume) as indicated by the arrows. The tip of the injection catheter was inserted close to the right atrium. Approximately 15 min elapsed between any two injections. 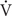
R, respiratory flow; VT, tidal volume; ABP, arterial blood pressure.

**FIGURE 2:**
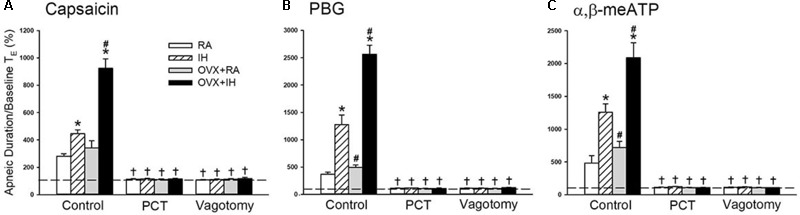
Role of lung vagal C fibers in IH-induced augmented apneic responses to stimulants in female rats. Groups of intact rats were exposed to RA or IH for 14 days, and OVX rats were exposed to RA (OVX + RA) or IH (OVX + IH) for the same duration. Each rat’s apneic responses to intravenous capsaicin **(A)**, PBG **(B)**, and α,β-meATP **(C)** were measured under control conditions, after perivagal capsaicin treatment (PCT; 250 μg/ml), and after vagotomy. Apneic duration indicates the longest expiratory duration (TE) during the first 20 s after stimulant injection. Baseline TE was calculated as the average over 10 consecutive breaths immediately before injection. The horizontal dashed lines indicate the apneic ratio of 100% (no response). ^∗^*p* < 0.05 compared with responses of RA rats under the same experimental condition; ^#^*p* < 0.05 compared with responses of IH rats; ^†^*p* < 0.05 compared with control responses in the same group. Except for the RA group, which included rats in the four stages of the estrus cycle (*n* = 6/each stage; total number of RA rats: 24), all data from the other groups are presented as means ± SE of 10 rats. See the legend of **Figure 1** for further explanation.

**FIGURE 3:**
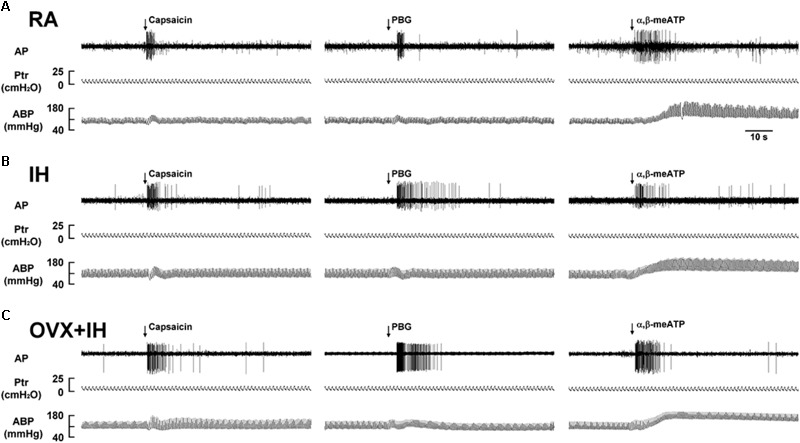
Responses of lung vagal C fibers to intravenous injection of three stimulants in three female rats exposed to RA or IH. **(A)** Responses of an intact rat exposed to RA; **(B)** responses of an intact rat exposed to IH; **(C)** responses of an OVX rat exposed to IH (OVX + IH). Afferent responses to capsaicin, PBG, and α,β-meATP were measured. These stimulants were injected into the jugular vein as a bolus (0.1 ml volume), as indicated by the arrows. Approximately 15 min elapsed between any two injections. AP, action potential; Ptr, tracheal pressure; ABP, arterial blood pressure. See the legend of **Figure 1** for further explanation.

When an RA-exposed diestrus female rat was investigated, intravenous capsaicin induced a mild respiratory inhibition that was characterized by apnea manifesting as prolonged TE (**Figures 1A, 2A**). No significant difference was found in the average apneic reflex responses to the same dose of capsaicin during the four stages of the estrus cycle, including proestrus, estrus metestrus, and diestrus (**Table 1**). Similar results were observed when phenylbiguanide and α,β-methylene-ATP were used individually as chemical stimulants (**Table 1**). In addition, the peak responses of LVCFs induced by chemical stimulants were measured to study LVCF responses. The intravenous injection of capsaicin, phenylbiguanide and α,β-methylene-ATP all triggered an abrupt discharge burst in an RA-exposed diestrus female rat (**Figure 3A**). The LVCF response evoked by any one of the three chemical stimulants was not significantly different from that of rats in the four stages of the estrus cycle (**Table 1**).

**Table 1:** Average apneic reflex responses and peak responses of lung vagal C fibers to chemical stimulants in intact female rats at the four stages of the estrus cycle upon exposure to RA.

Estrus stages	Proestrus	Estrus	Metestrus	Diestrus
**Apneic reflex responses to stimulants: apneic duration/baseline *T*_E_ (%)**				
Capsaicin	319.0 ± 42.6	267.8 ± 27.3	265.4 ± 27.3	290.7 ± 25.1
Phenylbiguanide	481.0 ± 72.7	318.3 ± 35.0	335.7 ± 41.4	450.0 ± 61.1
α,β-methylene-ATP	645.0 ± 96.2	398.7 ± 78.2	482.3 ± 43.0	472.8 ± 45.9
**Peak responses of LVCFs to stimulants (impulses/s)**		
Capsaicin	6.75 ± 0.50	5.88 ± 0.60	6.13 ± 0.55	6.33 ± 0.58
Phenylbiguanide	7.17 ± 0.71	7.50 ± 0.89	6.18 ± 0.97	6.67 ± 1.04
α,β-methylene-ATP	7.08 ± 0.61	6.75 ± 0.96	6.50 ± 1.84	7.06 ± 0.82

**FIGURE 4:**
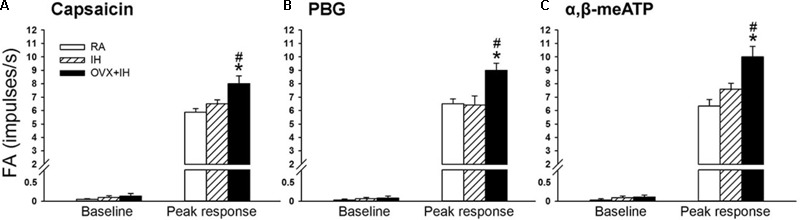
Sensitizing effect of IH on afferent responses of lung vagal C fibers to intravenous stimulants in female rats. Groups of intact rats were exposed to RA or IH for 14 days, and OVX rats were exposed to IH (OVX + IH) for the same duration. The afferent responses (fiber activity, FA) to intravenous capsaicin **(A)**, PBG **(B)**, and α,β-meATP **(C)** were measured. Baseline FA was calculated as the value averaged over 10-s interval before stimulation. The peak response was measured as the maximum averaged over 2-s interval after stimulation. Except for the RA group, which included rats in the four stages of the estrus cycle (*n* = 6/each stage; total number of RA rats: 24), all data from the other groups are presented as means ± SE of 10 fibers recorded from 10 rats. ^∗^*p* < 0.05 compared with peak responses of RA rats; ^#^*p* < 0.05 compared with peak responses of IH rats. See the legend of **Figure 1** for further explanation.

*Measurements were made in female rats at the four stages of the estrus cycle; *n* = 6 in each stage. Apneic reflex responses to capsaicin, phenylbiguanide, and α, β-methylene-ATP were separately observed in spontaneously breathing rats, while the peak responses of LVCFs to these stimulants were observed in additional artificially ventilated rats. Each rat was used for one estrus stage. Values are presented as means ± SE. LVCFs, lung vagal C fibers. See legends in **Figures 2, 4** for details. No significant differences were detected among rats in the four stages of the estrus cycle in response to the same stimulant.*

### Serum Levels of Estradiol in Rats of Different Estrus Stages and OVX Rats With Various E2 Replacements

**Table 2** shows the serum levels of estradiol in intact RA rats in the four stages of the estrus cycle. Estradiol concentration was higher in the proestrus stage than in the other three stages. The serum estradiol levels of OVX + IH rats supplemented with E2 at 30 and 150 μg/ml were similar to those in diestrus and proestrus stages in intact RA rats, respectively.

**Table 2:** Serum estradiol levels of various study groups.

Estradiol concentration (pg/ml)				
**Intact female rats**	**Estrus stages**			

	Diestrus	Proestrus	Estrus	Metestrus
	9.8 ± 0.7	33.4 ± 3.9	7.2 ± 0.3	4.5 ± 0.2

**OVX rats**	**E2 replacement**			

	Vehicle	30 μg/ml	50 μg/ml	150 μg/ml
	5.1 ± 0.4	9.4 ± 0.9	15.4 ± 1.2	24.7 ± 2.6

*Intact female rats were exposed to 14 days of RA (intact female rats) and OVX rats were exposed to 14 days of IH (OVX rats). Serum estradiol levels were measured in intact rats in the four estrus stages and in OVX rats supplemented with various concentrations of 17 β-estradiol (E2). Data of intact female rats at each estrus stage and OVX rats with different concentrations of E2 replacement are presented as the means ± SE of 6 and 10, respectively.*

### Role of LVCFs in OVX-Induced Augmented Apneic Response to Chemical Stimulants in IH-Exposed Rats

Given that different estrus stages did not significantly alter the apneic reflex responses and afferent responses of LVCFs to chemical stimulants in intact RA rats, the data from these groups were pooled. In addition, the apneic reflex responses to any one of these stimulants in OVX + RA rats was similar to those of RA rats; no significant difference was observed in the apneic responses to stimulants between RA and OVX + RA rats (**Figure 2**). The apneic responses to capsaicin (**Figure 2A**), phenylbiguanide (**Figure 2B**), and α,β-methylene-ATP (**Figure 2C**) were slightly but significantly increased in IH rats relative to those of RA rats. Furthermore, the same dose of three chemical stimulants elicited apneic responses in OVX + IH rats were significantly greater than those in all other groups (**Figures 1, 2**). Further analysis demonstrated that apneic responses to intravenous capsaicin, phenylbiguanide, and α,β-methylene-ATP were completely blocked by perivagal capsaicin treatment or bilateral vagotomy (**Figure 2**). Hence, these responses were reflexes mediated through LVCFs.

### Potentiating Effect of OVX on LVCF Responses to Chemical Stimulants in IH Rats

Considering that LVCFs mediate IH-induced augmented apneic responses, we then measured the LVCF activity induced by chemical stimulants. In an RA rat, the intravenous injection of capsaicin, phenylbiguanide, or α,β-methylene-ATP evoked a mild short burst of discharge (**Figure 3A**). The LVCF activities in RA and IH rats exhibited similar responses to capsaicin, phenylbiguanide, or α,β-methylene-ATP (**Figures 3A,B**). The baseline activities of the LVCFs or the average peak responses of the LVCFs to any one of these stimulants did not significantly differ between RA and IH rats (**Figure 4**). By contrast, the mean peak responses of the LVCFs evoked by capsaicin (**Figures 3C, 4A**), phenylbiguanide (**Figures 3C, 4B**), and α,β-methylene-ATP (**Figures 3C, 4C**) in OVX + IH rats were significantly greater than those in RA and IH rats.

### Role of Estrogen in the Potentiating Effect of OVX in IH Rats

**FIGURE 5:**
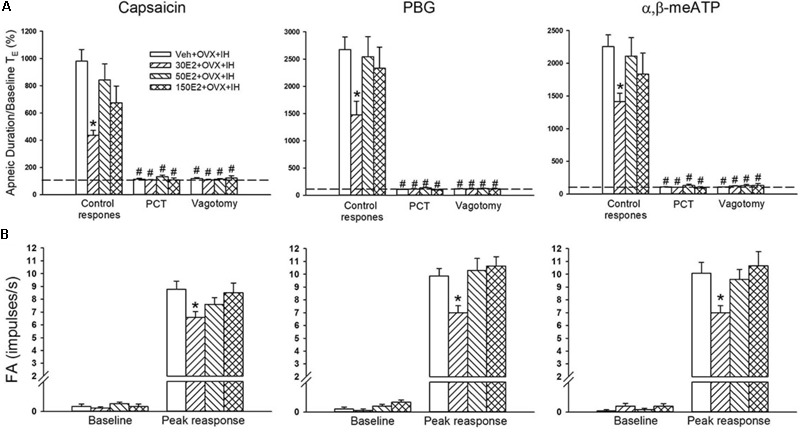
Role of estrogen in the potentiating effect of OVX on apneic responses and afferent responses to stimulants in female rats exposed to IH. Apneic responses **(A)** and afferent responses **(B)** to intravenous capsaicin, PBG, and α,β-meATP were measured after 14 days of exposure to IH in OVX rats supplemented with vehicle (Veh + OVX + IH) or three different concentrations of 17β-estradiol (E2): 30, 50, and 150 μg/ml (30E2 + OVX + IH, 50E2 + OVX + IH, 150E2 + OVX + IH). ^∗^*p* < 0.05 compared with Veh + OVX + IH rats; ^#^*p* < 0.05 compared with control responses in the same group. Data in each group are presented as means ± SE of 10 rats. See legend of **Figures 2** and **4** for further explanation.

Considering that estrogen may modulate airway hypersensitivity in females, we assessed the roles of supplementation with three different concentrations of E2 in the potentiating effect of OVX in IH rats. Reflex studies showed that in OVX + IH rats, augmented apneic responses to capsaicin, phenylbiguanide, and α,β-methylene-ATP were drastically attenuated by E2 supplementation at a low concentration (30 μg/ml) but not by vehicle supplementation (**Figure 5A**). Similarly, electrophysiological studies showed that the augmented peak responses of LVCFs to three chemical stimulants in OVX + IH rats were significantly attenuated by E2 supplementation at a low concentration but not by vehicle supplementation (**Figure 5B**). By contrast, E2 supplementation at high concentrations (50 and 150 μg/ml) had no significant influence on the augmented apneic responses (**Figure 5A**) and peak responses of LVCFs (**Figure 5B**) to chemical stimulants in OVX + IH rats.

### Role of Estrogen in Enhanced Lung Inflammation in OVX Rats Exposed to IH

**FIGURE 6:**
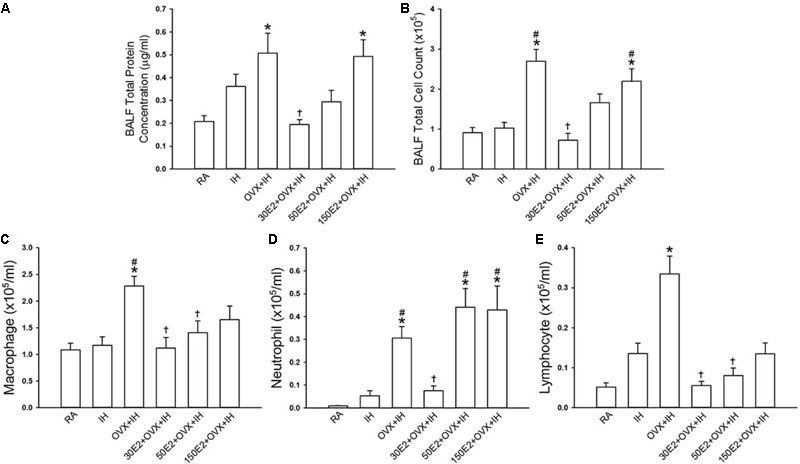
Changes in total cell count and differential cell counts in bronchoalveolar lavage fluid (BALF) from female rats. Two groups of intact rats were exposed to RA or IH for 14 days. The four other groups consisted of OVX rats alone (OVX + IH) or under supplementation with three different concentrations of E2: 30, 50, and 150 μg/ml (30E2 + OVX + IH, 50E2 + OVX + IH, 150E2 + OVX + IH) and exposed to IH for 14 days. The indices measured were total protein concentration **(A)**, total cell count **(B)**, and differential cell count: macrophage **(C)**, neutrophil **(D)**, and lymphocyte **(E)** in BALF sampled from rats of the six study groups. ^∗^*p* < 0.05 compared with responses of RA rats; ^#^*p* < 0.05 compared with responses of IH rats; ^†^*p* < 0.05 compared with OVX + IH rats. Data in each group are presented as means ± SE of five rats.

Estrogen plays a critical role in modulating inflammatory signaling pathways and inflammatory cell infiltration in lungs (Keselman and Heller, 2015). IH exposure did not significantly alter inflammatory cell infiltration in BALF in terms of total protein concentration and total number of leukocyte cells in intact rats (**Figure 6**). However, the BALF levels of total protein, total cell count, and differential cell counts, such as macrophages, neutrophils, and lymphocytes, in OVX + IH rats were significantly higher than those in RA and IH rats (**Figure 6**). The OVX combined with IH exposure-induced increase in inflammatory cell infiltration was not observed in OVX + IH rats under E2 replacement at a low concentration (30 μg/ml), but was unaffected by E2 replacement at a high concentration (150 μg/ml) (**Figure 6**).

**FIGURE 7:**
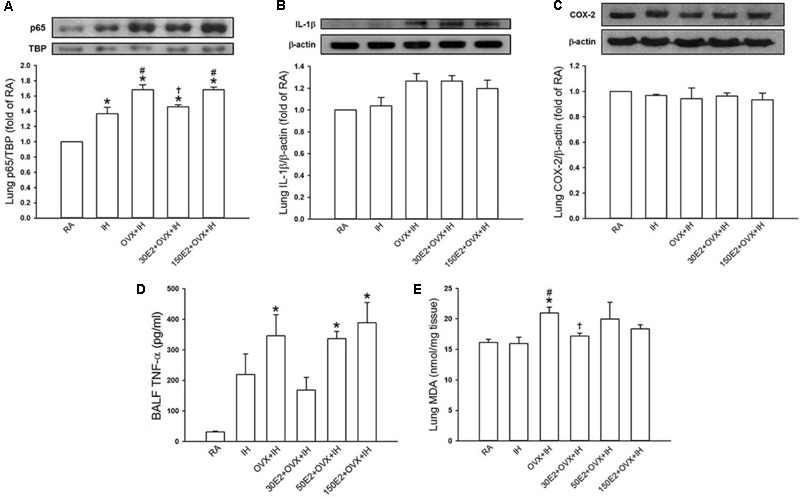
Expression of inflammatory cytokines and cyclooxygenase-2 (COX-2) and level of malondialdehyde (MDA) in lung samples and BALF from female rats. Two groups of intact rats were exposed to RA or IH for 14 days. The four other groups consisted of OVX rats (OVX + IH) or OVX rats receiving E2 replacement at the concentrations of 30, 50, and 150 μg/ml (30E2 + OVX + IH, 50E2 + OVX + IH, 150E2 + OVX + IH) and exposed to IH for 14 days. In **(A)**, the levels of the protein expression of the p65 subunit of NF-κB in the nucleus of lung tissue samples were analyzed through Western blot analysis to assess NF-κB activation. The protein levels of interleukin-1β [IL-1β **(B)**] and COX-2 **(C)** in the lung tissues were measured. The BALF level of TNF-α **(D)** and the lung level of MDA **(E)**, an indicator of oxidative stress, in these groups were analyzed through ELISA and an assay kit, respectively. These indices were measured and served as indications of lung inflammation. ^∗^*p* < 0.05 compared with responses of RA rats; ^#^*p* < 0.05 compared with responses of IH rats; ^†^*p* < 0.05 compared with OVX + IH rats. Data in each group are presented as means ± SE of five rats.

Biochemical analysis revealed that the lung protein levels of the p65 subunit of NF-κB in the nucleus were significantly higher in IH rats and OVX + IH rats than in RA rats (**Figure 7A**). Further analysis demonstrated that OVX combined with IH exposure exerted a greater effect on the elevated level of nuclear NF-κB-p65 than IH alone (**Figure 7A**). By contrast, no difference was found in the protein levels of IL-1β and COX-2 in lung tissues among all the groups (**Figures 7B,C**). In addition, the BALF level of TNF-α (**Figure 7D**) and lung level of lipid peroxidation (**Figure 7E**) in OVX + IH rats were significantly greater than those in the RA and IH rats. In OVX + IH rats, these augmented inflammatory responses were significantly attenuated by E2 supplementation at a low concentration (30 μg/ml) but was unaffected by E2 supplementation at a high concentration (150 μg/ml) (**Figures 7A,D,E**).

## Discussion

This study demonstrates that compared with RA exposure, 14 days of IH exposure slightly increased LVCF-mediated apneic responses and LVCF fiber responses to intravenous injection of capsaicin, phenylbiguanide, and α,β-methylene-ATP in intact anesthetized female rats. OVX combined with IH exposure significantly augmented reflex apneic responses and LVCF responses to the chemical stimulants. LVCF hypersensitivity in OVX rats exposed to IH was associated with lung inflammation as shown by the elevated infiltration of inflammatory cells (macrophages, neutrophils, and lymphocytes) in BALF, lung lipid peroxidation, and expression of inflammatory cytokines (NF-κB and TNF-α). Moreover, augmented apneic reflex and afferent responses to chemical stimulants and lung inflammation in OVX rats exposed to IH were markedly attenuated by E2 replacement at a low concentration (30 μg/ml) but not at high concentrations (50 and 150 μg/ml). All these results indicate that endogenous estrogen may protect against the development of LVCF hypersensitivity in female rats exposed to IH.

We previously found that in male rats, LVCF sensitivity can be enhanced by acute IH (Shen et al., 2012), 24 h of IH exposure (Yang et al., 2016a), and 14 days of IH exposure (Yang et al., 2016b). However, we did not determine whether female rats exhibit similar effects. Investigators have mostly focused on the effect of IH exposure on the pathogenesis of cardiorespiratory consequences in males and not in females. The prevalence of OSA is low in premenopausal women but is similar between post-menopausal women and men (Nieto et al., 2000; Jordan and McEvoy, 2003; Resta et al., 2003). Furthermore, the adverse effects of IH exposure or continuous hypoxia are less dramatic in females than in males (Ou et al., 1994; Hinojosa-Laborde and Mifflin, 2005) and are attenuated by female reproductive hormones, particularly estrogen (Ou et al., 1994). On the other hand, fluctuations in estrogen levels during the estrus cycle can modulate afferent sensitivity; for example, elevated sensitivity of the trigeminal sensory system to cutaneous stimuli was found in proestrus stage of rat estrus cycle (Martin et al., 2007). However, the apneic reflex and LVCF afferent responses to chemical stimulants were weakly elevated in RA rats in proestrus stage but did not significantly differ in rats in different stages of the estrus cycle (**Table 1**). The contradictory results of the previous study and this study may reflect the innervation of different target organs by different afferents. On the other hand, in the present study, the apneic responses to chemical stimulants and ABP in OVX + RA rats were similar to those in RA rats. One consideration for the results is that the 21-day period after OVX is probably insufficient to affect LVCF sensitivity and ABP under normal conditions. This possibility is supported by previous studies showing that elevated levels of inflammatory markers in heart tissues were found in rats 9 weeks after OVX (Hamilton et al., 2008) but not in mice 33 days after OVX (Torres et al., 2014).

Our previous study (Yang et al., 2016b) showed that the sensitivity of LVCFs to chemical stimulants drastically increased in male rats after 14 days of IH exposure. However, the current experiments showed that compared with RA exposure, 14 days of IH exposure did not significantly increase the afferent responses of LVCFs to chemical stimulants in intact female rats although IH exposure mildly enhanced apneic responses to stimulants (**Figures 2, 4**). The absence of the potentiating effect of IH on LVCF sensitivity in intact female rats may be due to an involvement of female reproductive hormones. Furthermore, our results demonstrated that reflex apneic responses and LVCF responses to stimulants in OVX + IH rats were significantly greater than those in all other groups (**Figures 2, 4**), suggesting that ovarian hormones exert a protective effect. This concept is further supported by previous studies showing that intact female animals are protected from IH-induced hypertension (Hinojosa-Laborde and Mifflin, 2005) and impairment of spatial learning and memory (Aubrecht et al., 2015); however, OVX removes this protection. The exact mechanism underlying the potentiating effect of OVX combined with IH exposure on LVCF sensitivity is unknown. One possibility is that endogenous estrogen may modulate lung inflammatory processes evoked by IH exposure. These processes, in turn, can participate in the development of LVCF hypersensitivity. Our recent laboratory work indicated that lung inflammation involving the release of ROS and COX metabolites is a major contributor to the development of airway hypersensitivity in IH-exposed male rats (Yang et al., 2016a,b). To investigate the actions of estrogen on the lung inflammation in IH-exposed female rats, we measured the levels of several inflammatory markers. Elevated levels of inflammatory markers, such as ROS, NF-κB, TNF-α, IL-1β, and COX metabolites in patients with OSA and IH-exposed animals have been reported (Kent et al., 2011; Yang et al., 2016a; Fernandez-Julian et al., 2017). Furthermore, IH can induce the transformation of monocytes to activated macrophages (Li R.C. et al., 2011), which can further react to promote the expression of various cytokines in the lungs (Gonzalez et al., 2007). In addition, neutrophil infiltration in the lungs is controlled by a complex network of chemokines and can induce ROS production (Boyle et al., 2012). Excessive ROS production is responsible for the development of cardiorespiratory consequences in patients with OSA and IH-exposed animals (Dhalla et al., 2000; Sugiura and Ichinose, 2008). Our results showed no significant differences in inflammatory cell infiltration in BALF and lung lipid peroxidation between RA and IH rats; however, the effect of IH on the development of inflammation was pronounced in OVX females (**Figures 6, 7E**). We believe that ovarian hormones attenuate lung inflammation in female rats exposed to IH and prevent LVCF hypersensitivity.

NF-κB is a critical regulator that induces the inflammatory cascade and is an inflammatory cytokine that triggers the pathophysiological responses in patients with OSA (Ryan et al., 2005) and in animal models of IH (Li S. et al., 2011). The inactivity of cytoplasmic NF-κB under resting conditions results from corepression by IκB. Upon stimuli, such as increased ROS production (Qian et al., 2012) or hypoxia exposure (Patel et al., 2017), IκB is phosphorylated and allows p50 and p65 to form the NF-κB heterodimer, which then translocates to the nucleus, resulting in the transcription of several target genes, such as TNF-α, IL-1β, and COX-2 (Ghosh et al., 1998). TNF-α can exert multiple biological effects on different cells and produce various inflammatory cytokines in the lungs (Lin et al., 2013), which has been considered a marker of the severity of OSA (Vgontzas et al., 2004). Moreover, TNF-α or IL-1β activates NF-κB (Sakon et al., 2003; Rius et al., 2008). Thus, the interplay among cytokines can elicit a positive regulatory loop, which in turn further amplifies inflammatory reactions. Inflammatory mediators and cytokines, including ROS, TNF-α, IL-1β, and COX-2 metabolites, are known to stimulate and/or sensitize LVCFs (Ho et al., 2000; Yu et al., 2007; Ruan et al., 2014; Lin et al., 2017). Our biochemical data showed that lung tissues collected from IH rats had slightly increased levels of nuclear NF-κB-p65 (**Figure 7A**). In addition, 14 days of IH exposure may be insufficient for inducing significant changes in the lung levels of IL-1β, COX-2, and TNF-α in intact rats. By contrast, OVX combined with IH exposure up-regulated the expression of lung inflammatory markers, including NF-κB and TNF-α (**Figure 7**). Similarly, LVCF-mediated apneic and LVCF afferent responses to chemical stimulants were markedly augmented in OVX + IH rats compared with those in IH rats (**Figures 2, 4**). Although we did not attempt to identify any specific cytokine that is mainly responsible for these effects, it seems reasonable to postulate that ovarian hormones may effectively prevent lung inflammation in intact rats exposed to IH; these events can mitigate the development of LVCF hypersensitivity.

Endogenous estrogen plays a protective role in the pathogenesis of chronic inflammatory diseases in females; for example, the levels of inflammatory cytokines were elevated in post-menopausal women and rats with OVX-induced E2 deficiency (Straub, 2007). Estrogen is also an antioxidant that stimulates the synthesis of antioxidant enzymes mediated by estrogen receptors (Vina et al., 2013). However, estrogen has a paradoxical role in the pathogenesis of chronic inflammatory diseases (Straub, 2007). For example, estrogen plays an pro-inflammatory role by activating inflammatory cells, such as mast cells and eosinophils (Hamano et al., 1998; Jensen et al., 2010) and an anti-inflammatory role by reducing the production of inflammatory cytokines (Kramer et al., 2004) and protecting cells from harmful oxidative stress (Strehlow et al., 2003). The contradictory role of estrogen in inflammatory processes is likely due to different stimuli, cell types, target organs, estrogen receptor expression, and estrogen concentrations (Straub, 2007). Interestingly, we found that the potentiating effects of OVX combined with IH on apneic reflex responses and LVCF sensitivity to chemical stimulants were absent in rats receiving E2 at a low concentration (30 μg/ml; diestrus level) (**Figure 5**) and the accompanying lung inflammation was abrogated (**Figures 6, 7**), but not in those receiving E2 at high concentrations (50 and 150 μg/ml). The possible role of estrogen in IH-induced airway hypersensitivity in female rats is concentration dependent. Specifically, in the IH-induced development of lung inflammation, estrogen plays a protective role at low levels but a detrimental role at high levels. In support of such assumptions, previous reports have indicated that low concentrations of E2 at the nanomolar level inhibit NF-κB activity mediated by estrogen receptor α (Kalaitzidis and Gilmore, 2005). By contrast, high E2 concentrations promote inflammatory responses (Hirano et al., 2007). Several studies have provided diverse explanations for the contradictory modulatory effects of the physiological concentrations of estrogen on inflammatory reactions (Straub, 2007; Draijer et al., 2016). In addition, the new findings provided by this study do not rule out the possible involvement of the enhancement of sensitivity and/or overexpression of estrogen receptors in the immune system and airway structural cells in females exposed to IH.

Increasing evidence has shown that OSA is associated with asthma (Teodorescu et al., 2012). LVCF hypersensitivity is a major contributor to the pathophysiology of asthma (Widdicombe, 2003). Epidemiological studies have documented the gender difference in the prevalence of asthma in different life stages (Ticconi et al., 2013). At younger age, the prevalence of asthma is higher in males but then becomes higher in females reaching the reproductive age (Ticconi et al., 2013). Furthermore, in female asthmatic patients, both preovulatory and perimenstrual phases may serve as triggers of acute asthma exacerbation (Brenner et al., 2005). All these observations suggest that female reproductive hormones, particularly estrogen, may be important factors influencing the development of hyperreactive airway diseases and its severity in humans (Ticconi et al., 2013). In addition to estrogen, the other additional ovarian hormones, such as progesterone, androgen, and several peptides, may have a functional role in these responses in females. During the physiological estrus cycle, the secretion profiles of these ovarian hormones correspond to the cyclic fluctuation of estrogen; moreover, these hormones play a synergistic role with estrogen to produce physiological functions (Adashi, 1994). In the present study, we found that some of these hormones may counteract estrogen in the development of LVCF hypersensitivity and lung inflammation following IH exposure. These observations may explain the lack of the potentiating effect of IH on LVCF sensitivity and lung inflammation in intact females, but OVX + IH rats receiving E2 at a high concentration (150 μg/ml; proestrus stage) showed the enhancement of these responses.

In summary, we demonstrated that OVX rats exposed to IH for 14 days displayed more severe lung inflammation than intact rats exposed to RA or IH, as evidenced by their increased inflammatory cell infiltration in BALF, lung lipid peroxidation, and protein expression of inflammatory cytokines. These effects were accompanied by the enhanced sensitivity of LVCFs to chemical stimulants. E2 replacement at a low concentration (30 μg/ml; diestrus level) but not at high concentrations (50 and 150 μg/ml) prevented LVCF hypersensitivity and lung inflammation in OVX + IH rats. Our findings provide novel insight on the pathogenic mechanisms of OSA-associated hyperreactive airway diseases in post-menopausal women.

## Author Contributions

Y-CH, ZFY, and CJL contributed to the concept and design of the research. Y-CH, C-HY, Y-JS, and J-YL performed the experiments. Y-CH, ZFY, C-HY, Y-JS, J-YL, and CJL analyzed and interpreted the data, edited and revised the manuscript. Y-CH, C-HY, Y-JS, and CJL prepared the figures.

## Conflict of Interest Statement

The authors declare that the research was conducted in the absence of any commercial or financial relationships that could be construed as a potential conflict of interest.
